# Decidual cell regulation of trophoblast is altered in pregnancies at risk of pre-eclampsia

**DOI:** 10.1530/JME-17-0243

**Published:** 2018-02-07

**Authors:** L B James-Allan, G S Whitley, K Leslie, A E Wallace, J E Cartwright

**Affiliations:** 1 Molecular and Clinical Sciences Research InstituteSt. George’s, University of London, London, UK; 2 Fetal Medicine UnitSt. George’s Hospital, London, UK

**Keywords:** decidua, stromal, trophoblast, pre-eclampsia, chemotaxis

## Abstract

Successful implantation and placentation are dependent on the interaction between decidual stromal cells (DSC) and extravillous trophoblast (EVT) cells. The extent of trophoblast invasion relies on communication between the placenta and maternal decidua. The cyclical process of decidualisation induces a transformation of endometrial fibroblasts to secretory DSC; these secreted products have many functions including the control of trophoblast invasion. Inadequate trophoblast invasion and remodelling of the uterine vessels (the spiral arteries) are associated with pregnancy disorders such as pre-eclampsia. Uterine artery Doppler resistance index (RI) in the first trimester of pregnancy can be used as a proxy measure of remodelling. DSC were isolated from pregnancies with normal (normal RI) or impaired (high RI) spiral artery remodelling. Following isolation, DSC were re-decidualised using cAMP and MPA and secretion of the decidualisation markers IGFBP-1 and prolactin assessed. We examined the impact of DSC-secreted factors on trophoblast cell function, using the EVT cell line SGHPL-4. We demonstrated that DSC exposed to decidual factors were able to re-decidualise *in vitro* and that the chemoattraction of trophoblasts by DSC is impaired in pregnancies with high RI. This study provides new insights into the role that DSC play in regulating EVT functions during the first trimester of pregnancy. This is the first study to demonstrate that DSC from pregnancies with impaired vascular remodelling in the first trimester secrete factors that inhibit the directional movement of trophoblast cells. This finding may be important in understanding aberrant trophoblast invasion in pregnancies where vascular remodelling is impaired.

## Introduction

During the mid-secretory phase of the menstrual cycle, the human endometrium becomes remodelled due to rising levels of post-ovulatory progesterone, in preparation for pregnancy. A major part of this process is the differentiation of oestrogen-primed spindle-shaped stromal cells, within the endometrium, into specialised secretory epithelial-like decidual cells. This process, termed decidualisation, begins around the terminal spiral arteries then subsequently occurs throughout the endometrium (de Ziegler *et al.* 1998). IGFBP1 and prolactin (PRL) are two of the main products secreted by the decidual stromal cells (DSC), and it is these that are primarily used as markers of decidualisation *in vitro.*

In humans, decidualisation occurs cyclically, independent of the presence of an implanting blastocyst. In the absence of a fertilised conceptus, the levels of progesterone fall in the late secretory phase of the menstrual cycle and the decidualised endometrial layer is shed, leading to menstruation. If a blastocyst implants successfully and pregnancy occurs, levels of progesterone remain high, the decidua is preserved and remodelling extends to the basal endometrial layer (Brosens *et al.* 2002). Decidualisation is critical for trophoblast invasion and placentation, demonstrated by various knock-out mouse models where removal of specific genes implicated in decidualisation, such as Hoxa10 and Src, lead to impaired decidualisation, implantation and often infertility (Lim *et al.* 1999, Shimizu *et al.* 2005, Lim & Wang 2010).

To maintain decidualisation *in vitro*, DSC are dependent on continuous stimulation from elevated intracellular levels of cAMP, sustained activation of the PKA pathway and the actions of progesterone (Gellersen & Brosens 2003). Progesterone and the cAMP/PKA pathway induce upregulation of downstream decidual transcription factors such as FOXO1, Hoxa10 and Hoxa11. These transcription factors are involved in regulating the promoters of the numerous factors that are secreted by DSC during decidualisation, including PRL and IGFBP1 (Kim *et al.* 2003, Godbole & Modi 2010).

During the first trimester of pregnancy, the placenta develops from the trophectoderm on the outer layer of the blastocyst. The invasive trophoblast cells lead implantation and invade in to the uterine epithelium, differentiating to form the villous structure of the placenta. Extravillous trophoblast (EVT) migrate from the villi attaching the placenta to the decidua. Two populations of invasive EVT exist; interstitial EVT that migrate and invade through the decidua to the inner third of the myometrium and endovascular EVT, which migrate into the lumen of uterine spiral arteries. EVT replace the arterial endothelial and vascular smooth muscle cells of the vessels transforming the normally small muscular arteries into distended flaccid vessels capable of high conductance (Pijnenborg *et al.* 1981). This creates a high-flow, low-resistance circulation, therefore increasing the blood flow to the foetus. DSC control trophoblast invasion to ensure there is adequate invasion in to the decidua; however, they can also act to limit excessive invasion, which could put the mother at risk (Graham & Lala 1991, Zhu *et al.* 2009).

From approximately 5 weeks of gestation invading EVT form plugs in the maternal spiral arteries occluding the arteries and preventing maternal blood flow entering the intervillous space, thereby protecting the placenta and foetus from oxidative damage (Jaffe & Woods 1993, Burton *et al.* 1999). Before 11 weeks of gestation, the partial pressure of oxygen in the placenta is significantly lower than that in the decidua and the decreased blood flow in the spiral arteries creates a physiological hypoxic condition (Rodesch *et al.* 1992, Jauniaux *et al.* 2001). From 12 to 13 weeks of gestation, the endometrial oxygen partial pressure values become lower than the placental values, demonstrating that at the end of the first trimester, the trophoblast plugs dislodge and maternal blood flows into the intervillous space from around 10 weeks of gestation (Rodesch *et al.* 1992, Jaffe and Woods 1993, Jauniaux *et al.* 2003). Consequently, comparisons can be made between less than and more than 10 weeks gestation (Robson *et al.* 2012, Wallace *et al.* 2015).

Inadequate trophoblast invasion and reduced spiral artery remodelling are associated with pre-eclampsia and foetal growth restriction (FGR) (Pijnenborg *et al.* 1991). The pathogenesis of pre-eclampsia occurs in the first trimester when the EVTs are migrating and invading through the decidua to remodel the spiral arteries, whereas the clinical symptoms do not manifest until the second to third trimester of pregnancy. Uterine artery Doppler ultrasound is a non-invasive tool to evaluate the blood flow in the uterine arteries during pregnancy. The RI in these vessels can be assessed and can be used as a proxy measure of spiral artery remodelling and to identify those pregnancies at a higher risk of pre-eclampsia and/or FGR. In the first trimester, pregnancies with a RI > 95th percentile present with less trophoblast invasion of spiral arteries and have a 5-fold higher risk of developing complications of pregnancy, such as pre-eclampsia and FGR (Prefumo *et al.* 2004, Leslie *et al.* 2015).

The extent of trophoblast invasion relies on communication between the placenta and maternal decidua. The decidua regulates the process of EVT invasion by the factors produced by DSC upon decidualisation, as well as contributions from the populations of decidual immune cells present. DSC secrete a variety of factors, including the decidual markers prolactin and IGFBP1, as well as cytokines, growth factors and interleukins (Gellersen & Brosens 2014). These factors have an autocrine role, amplifying and continuing decidualisation, and a paracrine role, regulating trophoblast and immune cell function.

In this study, we hypothesised that DSC from pregnancies with impaired spiral artery remodelling alter the functionality of EVT, therefore contributing to the reduced trophoblast invasion and consequent defective spiral artery remodelling observed in pre-eclampsia. To test this hypothesis, we used uterine artery Doppler ultrasound as a proxy measurement of normal or impaired spiral artery remodelling in women undergoing first trimester terminations of pregnancy. DSC were isolated and used to investigate the effect of first trimester DSC on EVT cell functions.

## Materials and methods

### Uterine artery Doppler ultrasound

Doppler ultrasound examination of the maternal uterine arteries was performed in women attending a clinic for termination of pregnancy in the first trimester. 71 women were recruited to the study. Inclusion criteria consisted of singleton pregnancies, gestational age of 8–14 weeks, normal foetal anatomical features, no known medical conditions or history of recurrent miscarriages. Gestational age was calculated from the last menstrual period and confirmed by crown-rump length measurement. Local ethical committee (ref: 01.78.5) approval was obtained for this study; all women gave informed written consent.

Doppler ultrasound scanning was carried out by a trained and accredited sonographer (Fetal Medicine Unit, St. George’s Hospital) as previously described (Hollis *et al.* 2001). The RI of the uterine arteries was calculated, as well as the presence or absence of an early diastolic notch. Measurements were obtained on the left and right side, and the mean RI was calculated. Pregnancies between 9 and 14 weeks of gestation with a mean RI above the 95th centile and bilateral uterine artery notches were defined as having a high uterine artery RI. Normal RI cases were defined as presenting with a mean RI below the 95th centile. High RI groups have a five-fold risk of developing pre-eclampsia compared to normal RI groups (Leslie *et al.* 2015). Pregnancies between 8 and 9 weeks of gestation with a pulsatility index of >3.4, in addition to the mean RI, were used to characterise pregnancies that had a higher risk of developing pre-eclampsia.

### DSC isolation

Decidual tissue isolated from the products of conception obtained at the termination of pregnancy was minced and digested in serum-free medium 199 containing 0.1 mg/mL DNase (Sigma Aldrich) and 2 mg/mL collagenase (10 kU; Gibco/ThermoFisher Scientific) overnight at room temperature with constant agitation. The digested decidua was filtered, centrifuged and resuspended in 2% (v/v) fetal calf serum in PBS (Biosera, East Sussex, UK), layered onto Ficoll-Pacque (GE Healthcare) and centrifuged for 20 min at 710 ***g***. The cells in the buffy layer were collected, centrifuged for 5 min at 500 ***g*** and incubated in red blood cell lysis buffer (155 mM ammonium chloride (VWR, Leicestershire, UK), 9.9 mM Trizma base (Sigma Aldrich), pH 7.4) at room temperature for 5 min and centrifuged for 5 min at 500 ***g***. Cells were resuspended in DSC cell culture medium (RPMI 1640 medium supplemented with 10% (v/v) fetal bovine serum (FBS), containing 2 mmol/L l-glutamine, 100 IU/mL penicillin, 100 μg/mL streptomycin and 2.5 μg/mL amphotericin) and plated in a 37°C incubator for 15 min. DSC adhered to the bottom of the plate. Purity of the isolated DSC was assessed by positive immunocytochemical staining of the fibroblast marker vimentin and negative expression of the trophoblast marker CK7. The isolated cells were 94.2% ± 4.3% (mean ± s.e.m., *n* = 3) vimentin positive and CK7 negative, therefore confirming that the isolation method provides a pure yield of DSC.

### Cell culture and re-decidualisation

Decidual stromal cells were cultured in DSC culture medium. Once confluent, after approximately 2–5 days, cells were stimulated with 10 mM medroxyprogesterone17-acetate (MPA; Sigma Aldrich) and 0.5 mM 8 bromocyclic AMP (cAMP; BioLog Life Science Institute, Germany) in FBS-free Hams F10 medium to induce a differentiated phenotype. The vehicle control was 10 mM chloroform in FBS-free Hams F10 medium.

DSC were treated for 72 h and conditioned media (CM) was collected and concentrated 20-fold (VivaSpin columns, 3000 mol wt. cutoff: Sartorius Stedium, Surrey, UK) for use in experiments.

The human extravillous trophoblast (EVT) cell line, SGHPL-4, was cultured in Hams F10 medium supplemented with 10% (v/v) FBS, containing 2 mmol/L l-glutamine, 100 IU/mL penicillin and 100 μg/mL streptomycin (Cartwright *et al.* 1999).

### ELISA

The concentration of IGFBP1 and PRL in DSC CM was measured using Human Duo-Set enzyme-linked immunosorbent assay (ELISA) kits (R&D Systems), according to the manufacturer’s instructions. Where the analyte reading was below the sensitivity of the assay, an arbitrary value of half of the lowest standard for that experiment was used. All R&D Systems Duo-Set ELISAs typically have coefficient of variation (CV) values less than 10% across the standard curve for both intra- and inter-assay precision.

### EVT motility, proliferation and apoptosis assays

The methods were adapted from previously described methods (Cartwright *et al.* 1999). SGHPL-4 cells were cultured at a concentration of 3.0 × 10^4^ cells/mL in SGHPL-4 medium. Cells were serum starved in 0.5% (v/v) FBS overnight. Re-decidualised DSC (rDSC) CM at 1× concentration or control media (Hams F10 medium containing 10 mM MPA and 0.5 mM cAMP) was added to the SGHPL-4 cells.

Analysis of motility, proliferation and apoptosis was performed by time-lapse microscopy using an Olympus IX70 inverted microscope with motorised stage and cooled charge-coupled device camera and enclosed in a heated and humidified chamber at 37°C with 5% CO_2_ in air. Images were taken every 15 min for 24 h. For motility, time-lapse sequences were analysed using ImageJ software, version 1.46r (NIH) with the Manual Tracking tool, version 1.01 plug-ins (NIH). For apoptosis and proliferation, time-lapse sequences were analysed using Image-Pro Insight software. Analysis of cell motility was measured by randomly choosing 10 cells in two fields of view, which were manually tracked over 24 h. The distance moved (arbitrary units) was recorded. Cell proliferation and apoptosis was measured by randomly choosing 20 cells in two fields of view per treatment, which were observed over 24 h. Cells were scored at the time point they divided to assess proliferation. Apoptosis was determined when a cell showed apoptotic morphology, including a phase bright appearance and membrane blebbing and blistering (Cartwright *et al.* 1999, Ashton *et al.* 2005).

### Chemotaxis assay

SGHPL-4 cell chemotaxis toward rDSC CM was measured using the μ-Slide Chemotaxis 2D assay (Ibidi, Martinsried, Germany), according to the manufacturer’s instructions and a previously described method (Wallace *et al.* 2013).

In brief, cultivation areas of µ-slide were coated in 60 µg/mL rat tail collagen Type 1 (BD Biosciences) in 0.02 M acetic acid. Each chamber on the µ-slide was seeded with 1.8 × 10^4^ cells and incubated for approximately 4 h to allow cells to adhere. rDSC or DSC control medium was pipetted into the upper and lower reservoirs of each chamber and DSC CM or rDSC CM applied to either the upper or lower reservoir.

Analysis of chemotaxis was performed by time-lapse microscopy using an Olympus IX70 inverted microscope, as described earlier. Images were taken every 15 min for 24 h, and time-lapse sequences were analysed using ImageJ software, version 1.46r with the Manual Tracking and Chemotaxis tool, version 1.01 plug-ins. Analysis of directional cell movement was measured by randomly choosing 20 cells in two fields of view, which were manually tracked over 24 h. The number of cells moving towards a chemotactic stimuli were recorded.

### Statistical analysis

Data were analysed by one-way analysis of variance (ANOVA) or *t*-test, as indicated, using GraphPad Prism (version 6.0, CA, USA). ELISA data were transformed by taking the log of the concentration to ensure equal variance.

## Results

### Isolated first trimester DSC secrete the decidual factors IGFBP1 and PRL when stimulated *in vitro*

**Figure 1:**
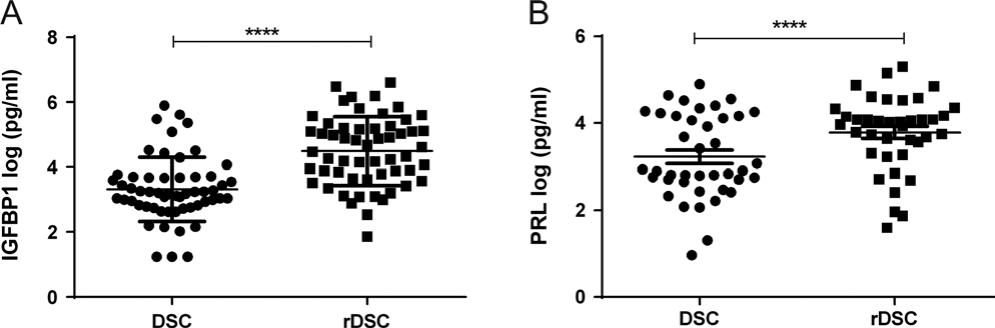
Isolated first trimester DSC expression of the decidual markers IGFBP1 and PRL. Isolated DSC were stimulated with cAMP and MPA (re-decidualised, rDSC) or a vehicle control (DSC) for 72 h. Conditioned media was collected and levels of decidual markers IGFBP1 and PRL measured by ELISA. Secretion of (A) IGFBP1 (*n* = 53) and (B) PRL (*n* = 40) was found to be significantly different between DSC and rDSC. Data were log-transformed to ensure equal variance. Paired *t*-test, data shown as mean ± s.e.m., ****P < 0.0001.

**Figure 2:**
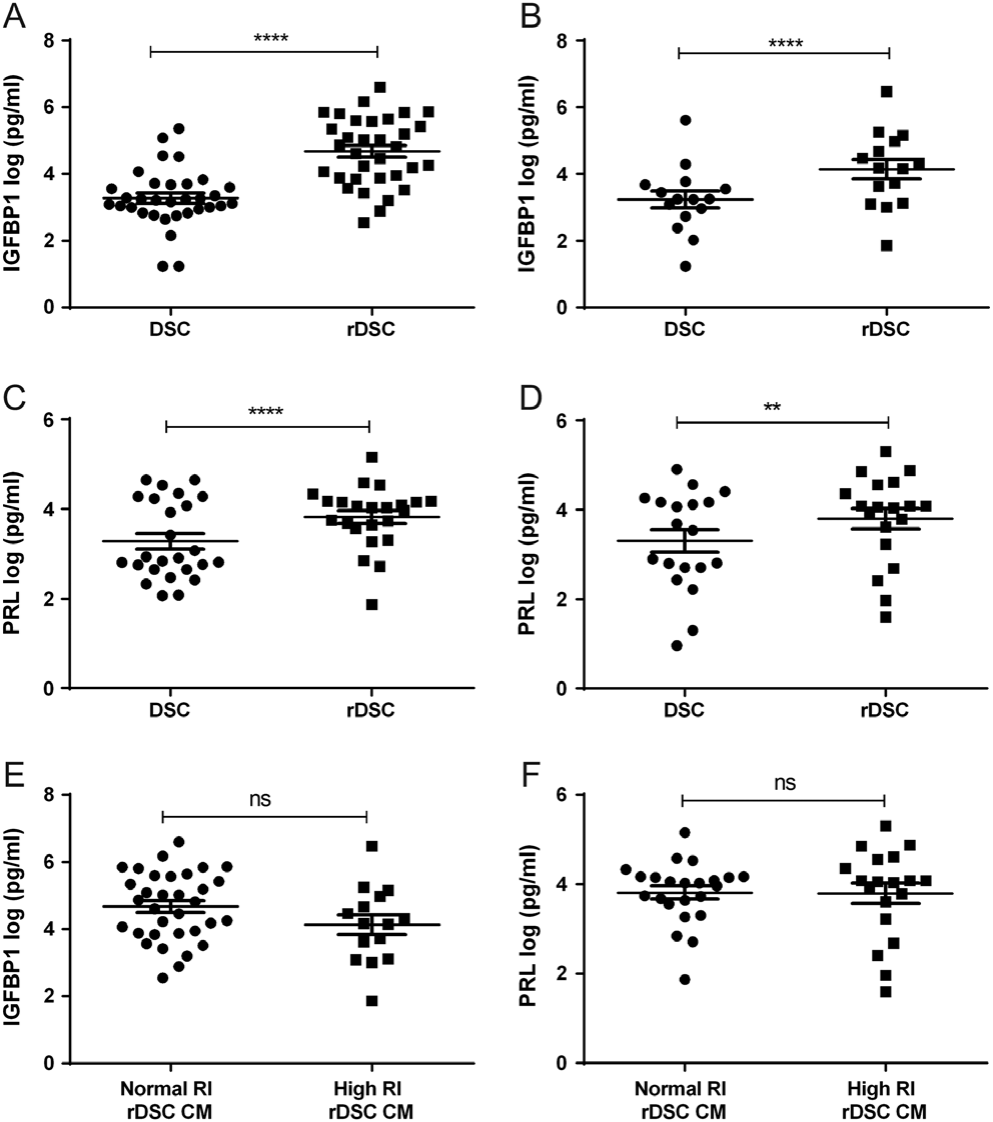
Secretion of IGFBP1 and PRL from normal resistance index (RI) and high RI DSC. Isolated DSC, from normal RI and high RI pregnancies, were stimulated with cAMP and MPA to stimulate decidualisation, after 72 h conditioned media was collected and levels of IGFBP1 and PRL measured by ELISA. Secretion of IGFBP1 was significantly increased in re-decidualised (rDSC) compared to DSC from normal RI (A) (*n* = 33) and high RI (B) (*n* = 15) pregnancies. Levels of PRL were significantly increased in rDSC compared to DSC CM from normal RI (C) (*n* = 25) and high RI (D) (*n* = 19) pregnancies. Secretion of (E) IGFBP1 or (F) PRL from rDSC from normal RI and high RI did not differ. Data were log transformed to ensure equal variance. Paired *t*-test (A, B, C and D), unpaired *t*-test (E and F), data shown as mean ± s.e.m., ****P < 0.001 and **P < 0.01, ns denotes not significant.

DSC isolated from uterine artery Doppler screened first trimester terminations of pregnancy were re-decidualised by stimulation with cAMP and the progesterone agonist MPA, to maintain their differentiated DSC phenotype *in vitro*. After 72 h of stimulation, CM from the cells was collected and re-decidualisation was assessed by the levels of the decidual markers IGFBP1 and PRL secreted by the cells compared to their non-decidualised control. DSC were able to differentiate to a decidualised phenotype, shown by increased levels of IGFBP1 (Fig. 1A) and PRL (Fig. 1B) in treated cells. Re-decidualised DSC from normal and high RI pregnancies both secreted increased levels of IGFBP1 (Fig. 2A and B) and PRL (Fig. 2C and D) compared to the untreated cells. There were no significant differences in re-decidualisation markers between normal and high RI DSC (Fig. 2E and F).

### Trophoblast motility, apoptosis and proliferation are not regulated by DSC

**Figure 3:**
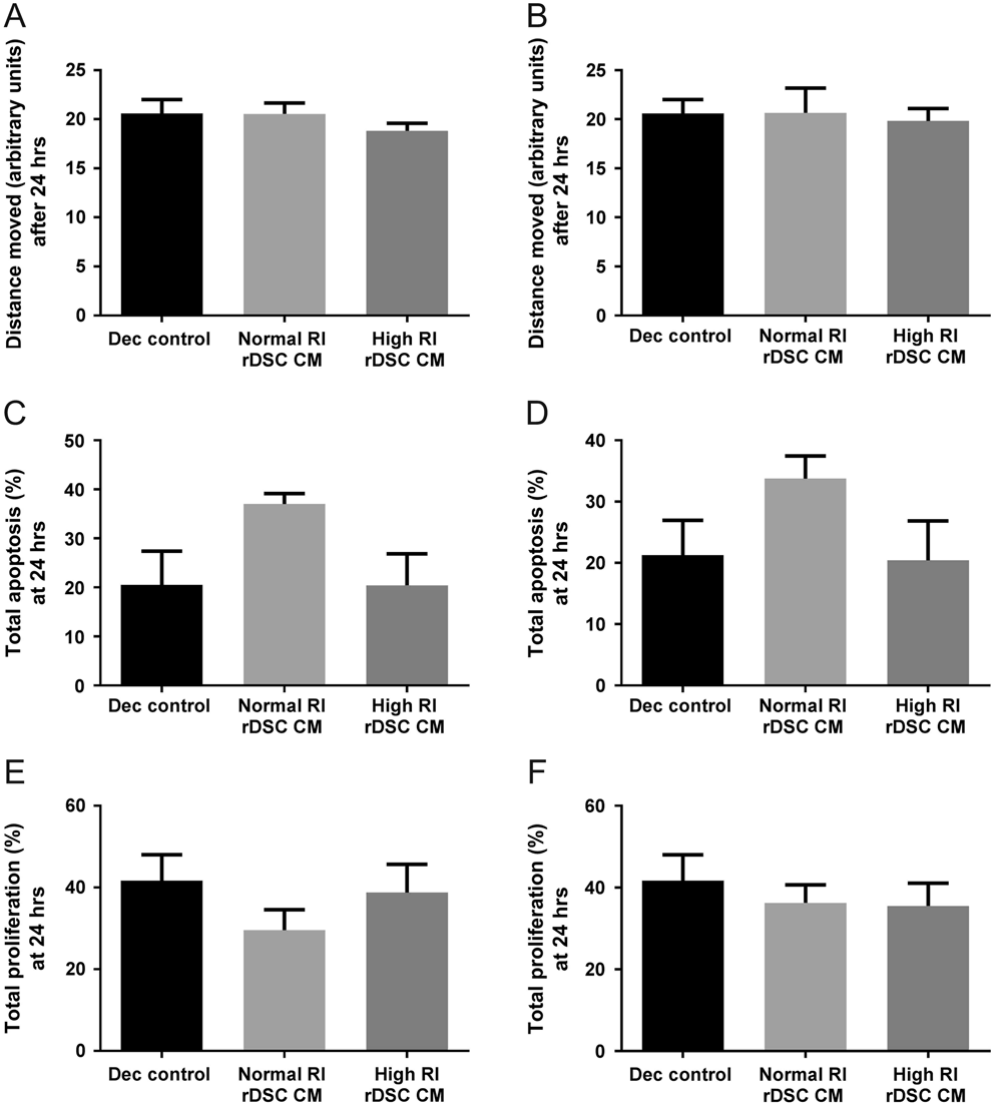
Effect of normal RI and high RI rDSC CM on SGHPL-4 cell motility, apoptosis and proliferation. The effect of re-decidualised (rDSC) CM from normal RI and high RI pregnancies on SGHPL-4 motility trophoblast motility (A: <10-week gestation, B: >10-week gestation), apoptosis (C: <10-week gestation, D: >10-week gestation) or proliferation (E: <10-week gestation, F: >10-week gestation) was assessed over 24** **h. Dec control is media with the decidualisation factors added (cAMP and MPA). No difference in motility, apoptosis or proliferation was observed when incubated with normal or high RI rDSC CM from <10 weeks and >10 weeks gestation (*n* = 6). ANOVA, data shown as mean + s.e.m.

SGHPL-4 cells, a well-characterised EVT cell line, were cultured with CM from DSC re-decidualised *in vitro* from normal and high RI pregnancies. The effect on SGHPL-4 motility, proliferation and apoptosis was measured over 24 h. No significant differences were observed in trophoblast motility (Fig. 3A: <10 weeks gestation, Fig. 3B: >10 weeks gestation), apoptosis (Fig. 3C: <10 weeks gestation, Fig. 3D: >10 weeks gestation) or proliferation (Fig. 3E: <10 weeks gestation, Fig. 3F: >10 weeks gestation) when incubated with CM DSC from normal or high RI pregnancies.

### Trophoblast chemotaxis is induced by DSC isolated from normal RI pregnancies but not high RI pregnancies at <10 weeks gestation

**Figure 4:**
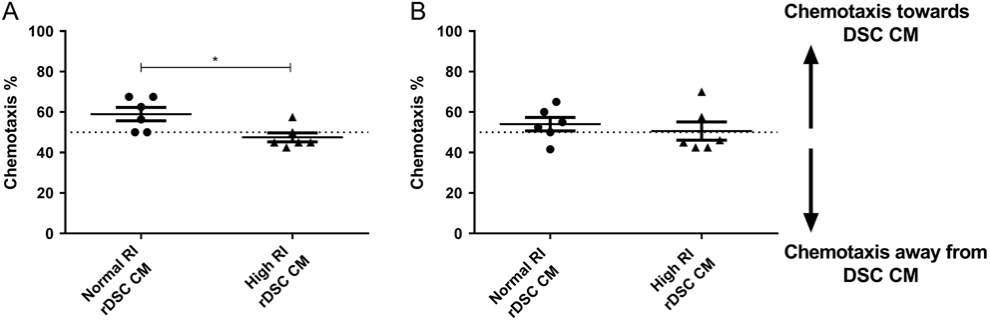
Effect of rDSC CM from normal and high RI pregnancies on chemotaxis of SGHPL-4 cells. SGHPL-4 cells were cultured in chemotaxis chambers to analyse chemotactic capacity of normal and high RI rDSC CM. Non-directional movement of cells gives expected chemotaxis of 50%. (A) Normal RI rDSC CM from pregnancies <10 weeks gestation is significantly more chemotactic to SGHPL-4 cells than high RI rDSC CM from pregnancies at <10 weeks gestation. (B) Normal and high RI rDSC CM from pregnancies >10 weeks gestation have no chemotactic effect on SGHPL-4 cells (*n* = 6). Paired *t*-test, data shown as mean ± s.e.m., *P < 0.05.

Trophoblast chemotaxis towards rDSC CM was examined using high and normal RI rDSC CM as a chemoattractant. Non-directional movement of the cells would be expected to produce 50% cell chemotaxis toward a chemoattractant. Using a positive control of media with 10% FCS the directional movement was 63.75% ± 2.1 (mean ± s.e.m., *n* = 24). When normal RI rDSC CM from pregnancies at <10 weeks gestation was used as a stimulus, 59% ± 3% (mean ± s.e.m., *n* = 6) of SGHPL-4 cells moved towards the CM. This was significantly more directional movement than SGHPL-4 cells to high RI rDSC CM, with 48% ± 2% (mean ± s.e.m., *n* = 6) of SGHPL-4 cells migrated toward the high RI rDSC CM (*P* < 0.05) (Fig. 4). There was no significant difference in migration of SGHPL-4 cells towards high or normal RI rDSC CM from pregnancies over 10-weeks gestation.

## Discussion

Decidualisation of the endometrium and the resulting DSC play a key role in implantation, placentation and therefore success of the pregnancy. However, the role that DSC play in the control of trophoblast function is still largely unknown. In this study, we have isolated DSC from first trimester terminations of pregnancy, and using uterine artery Doppler RI, where RI is a proxy measurement of spiral artery remodelling, have separated the DSC into normal RI and high RI groups (Prefumo *et al.* 2004). A high RI uterine artery Doppler measurement correlates to a 5-fold higher risk of developing pre-eclampsia, if the pregnancy were to continue (Leslie *et al.* 2015). Our study has shown significant differences in the secretion of decidualisation markers IGFBP1 and PRL in re-decidualised DSC, confirming that first trimester DSC can be re-decidulised in culture. We have demonstrated that the chemoattraction of trophoblast by rDSC is impaired in early gestation pregnancies with a high RI. Consequently, impaired chemoattraction of trophoblast cells in the first trimester of pregnancy, due to decidual secretions, could contribute to the reduced spiral artery remodelling observed in high RI pregnancies.

Re-decidualisation is necessary *in vitro* as isolated stromal cells lose their differentiated phenotype with time in culture and increased passage number (Brosens *et al.* 1999, Saleh *et al.* 2011). The decidual process is initiated and sustained through activation of the ubiquitous second messenger cAMP, which is enhanced by the actions of progestins (Brosens *et al.* 1999). The results of this study confirms that *in vitro* stimulation with cAMP and the synthetic progestin, MPA can differentiate isolated first trimester DSC to a re-decidualised phenotype.

Previous studies have demonstrated differences in the function and phenotype of trophoblast and decidual NK cells from the uterine artery Doppler-screened groups (Whitley *et al.* 2007, Fraser *et al.* 2012, Wallace *et al.* 2013, 2014, 2015). This study documents the first comparison of DSC isolated from first trimester pregnancies with impaired spiral artery remodelling, and therefore, an increased risk of pre-eclampsia, with DSC from normal RI pregnancies. Cyclic decidualisation, where decidualisation and menstruation occur monthly in the absence of pregnancy, has been termed ‘menstrual preconditioning’ to allow for embryo selection and deep placentation (Teklenburg *et al.* 2010). Aberrant decidualisation has been linked with clinical disorders, including endometriosis and recurrent pregnancy loss, and therefore, affects disorders where placentation is impaired, such as pre-eclampsia (Klemmt *et al.* 2006, Salker *et al.* 2012). This study suggests that DSC from pregnancies with an increased risk of developing pre-eclampsia are able to decidualise *in vitro.* However, there are additional markers of decidualisation, including LEFTY2, a NODAL signalling pathway inhibitor, which is highly expressed by decidualising cells, the key transcription factor CCAAT/enhancer-binding protein (C/EPB) and secreted products, such as Wnt5a, Dickkopf 1 (DKK1) and prokinectin (PROK1), which could be investigated further (Tabibzadeh *et al.* 1998, Christian *et al.* 2002, Matsuoka *et al.* 2010, Macdonald *et al.* 2011). An alternative interpretation of this data is that aberrant decidualisation does not play a key factor in the pathology of disorders such as pre-eclampsia. We have previously shown that trophoblast from high RI pregnancies are more sensitive to undergoing apoptosis (Whitley *et al.* 2007) and therefore the contribution of both the placental and decidual cells needs to be considered in partnership.

Decidualisation induces stromal cells to become secretory; in the first trimester, DSC produce a milieu of factors that influence trophoblast function (Sharma *et al.* 2016). As EVT invade from the tips of anchoring placental villi, they come into contact with this enriched decidual environment. In order to assess the influence that DSC have on EVT cells during the first trimester of pregnancy CM from DSC, isolated from normal and high RI pregnancies, were cultured with SGHPL-4 cells, an EVT cell line, and motility, proliferation and apoptosis were assessed. The results of this study demonstrate that secreted factors from first trimester decidualised DSC do not regulate trophoblast migration, proliferation or apoptosis *in vitro*, with no differences induced by DSC CM from normal and high RI pregnancies on SGHPL-4 cells.

Although non-directional EVT migration was not affected by DSC-secreted factors, invasion also consists of directional motility, termed chemotaxis and movement through a matrix. Gellersen *et al*. (2013) showed that chemotaxis of the invasive trophoblast cell line AC-1M88 was stimulated by the human endometrial stromal cell line T-HESC (Gellersen *et al.* 2013). As DSC *in vivo* secrete a range of chemokines, the effect of first trimester DSC-secreted factors on SGHPL-4 chemotaxis was investigated. We have demonstrated that DSC CM from normal RI pregnancies (but not high RI pregnancies) with a gestation of less than 10 weeks were able to stimulate chemotaxis of SGHPL-4 cells on collagen. However, this stimulation was lost with normal RI DSC CM from pregnancies more than 10 weeks gestation. This finding suggests that DSC-secreted factors stimulate the directional movement of EVT cells during the period they are migrating through the decidua towards the spiral arteries to plug these vessels. After 10 weeks of gestation, the EVT plugs begin to dissipate and therefore DSC-induced chemotaxis of EVT may no longer be necessary. Chemotaxis is most-likely caused by the secretion of potent chemokines by DSC, such as CXCL10 and IL-6, which have previously been shown to induce trophoblast cell chemotaxis (Dominguez *et al.* 2008, Sharma *et al.* 2016).

The results of this study indicate that DSC isolated from pregnancies with a high RI, indicating impaired spiral artery remodelling and an increased risk of pre-eclampsia, and normal RI both have the ability to be re-decidualised *in vitro*. Taken together, our results suggest that although secreted products from first trimester DSC do not regulate EVT motility, proliferation or apoptosis, DSC-secreted factors from high RI DSC inhibit EVT chemotaxis. Further investigation will highlight differences between normal and high RI DSC that may be contributing to impaired invasion and remodelling observed in pre-eclampsia.

## Declaration of interest

The authors declare that there is no conflict of interest that could be perceived as prejudicing the impartiality of the research reported.

## Funding

L B J-A was funded by a St. George’s, University of London studentship. A E W was supported by a grant from the Wellcome Trust (091550). Part of the research was supported by a grant from Action Medical Research UK (SP4577).
